# Correction

**DOI:** 10.1080/22423982.2024.2320471

**Published:** 2024-02-23

**Article title**: Factors associated with receiving an initial COVID-19 vaccine among Alaskan residents: results from an online cross-sectional survey

**Authors**: Gabriel Garcia, Jennifer Meyer, Alexandra Edwards and Drew Cameron

**Journal**: *International Journal of Circumpolar Health*

**Bibliometrics**: Volume 82, Number 1, pages 1-10

**DOI**: https://doi.org/10.1080/22423982.2023.2252604

It has been noted by the authors that the decimal for the percentages was misplaced in the fourth column for a couple of items in Table 2. The corrected table 2 placed below. This correction has not changed the description, interpretation, or original conclusions of the article. T&F apologises for any inconvenience caused.

**Table 2:** 

Demographic Characteristics			Alaska Population [1]
Sex	Frequency	Percent or Mean (S.D.)	Percent
Male	388	42.60%	52.40%
Female	523	57.40%	47.60%
Education			
< College degree	479	50.70%	69.40%
â‰¥ College degree	465	49.30%	30.60%
Race/Ethnicity			
Alaska Native/American Indian	115	12.10%	15.20%
Mixed and other racial/ethnic minorities [2]	159	16.80%	20.30%
Whites	639	67.40%	64.50%
Age			
Mean	948	50.41 (16.42) [3]	See note [4]
Annual Household Income			
< $80,000	411	45.80%	See note [5]
â‰¥ $80,000	486	54.20%	
Marital Status			
Not Married	531	44.00%	49.00%
Married	417	56.00%	51.00%
Political Ideology [6]			
Liberal	318	34.50%	23.00%
Moderate	340	36.80%	37.00%
Conservative	265	28.70%	34.00%
Alaska Region			
Anchorage	357	37.90%	39.70%
Gulf Coast	110	11.70%	11.10%
Interior	159	16.90%	14.00%
Matanuska-Susitna	120	12.70%	14.60%
Other (Northern, Southeast, Southwest) [7]	197	20.80%	20.60%
Ever Received COVID-19 Vaccine			
Yes	754	80.20%	66.3% [8]
No	186	19.80%	33.70%

